# 

**Published:** 1874-10

**Authors:** 


☞ This issue of the RECORD has been delayed a few days, owing to the press of business occasioned by the State Fair, the grandest success in that line known to the history of Georgia.

*To Our Subscribers.*--Our thanks are especially due those of our subscribers who have remitted their dues since the issue of the September number, and thus aided us in carrying out our contract with our publishers. Those who have not done us this favor, will please do so immediately after receiving this number, and thereby relieve us during the "hard times!" Don't fail, friends.

☞ Write your Names, Post Office, County and State *plainly*. Be sure to say what Post Office you wish the RECORD sent, and always date your letters. We receive many well written letters, but doubt even the authors being able to decipher their signatures.

☞ Address all Communications, to POWELL & GOLDSMITH, none other.

☞ List of Cash Payments will appear in due time.

☞ Send Money by Check, Postal Order or by Registered Letters. We are willing to endorse for the honesty of our subscribers, but cannot be responsible for the irregularity of the mails.

### ASSOCIATE EDITORS:

GEORGIA.

James A. Long, M.D.
W. L. M. Harris, M.D.
H. L Battle, M.D.
W. C. Musgrove, M D
L. B. Bouchelle, M.D.
Thomas Burdell, M.D.
E. H. W. Hunter, M.D.
V. S Hitt, M.D.
J. E. Pope, M.D.
A. H. Brantley, M.D.
J. J. Knott, M.D.
J. C. Wisdom, M.D.
W. W. Leake, M.D.
S. G. WHITE, M.D.
W. H. HALL, M.D.
G. D. CASE, M.D.

KENTUCKY.

W. S. Taylor, M.D.
B. W. Stone, M.D.
Charles Kearns, M.D.

TEXAS.

S. M. Welch, M.D.
T. J. Heard, M.D.
W. A. Heard, M.D.

TENNESSEE.

J. D. Tucker, M.D.

ALABAMA.

J. C. Knox, M.D.
Geo. F. Taylor, M.D.
J. B. Barnett, M.D.
S. M. Hogan, M.D.
D. S. Hopping, M.D.
J. T. Searcy, M.D.
S. F. Hill, M.D.

NORTH CAROLINA.

J. B. Hughes, M.D.
S. S. Satchwell, M.D.
A. B. Pierce, M.D.
H. Kelly, M.D.
Geo. A. Foote, M.D.
E. A. Anderson, M.D.
H. T. Bahnson, M.D.
Willis Alston, M.D.
Thos. F. Wood, M.D.
N. J. Pittman, M.D.
John W. Booth, M.D.

SOUTH CAROLINA.

E. T. McSwain, M.D.
G. M. Rivers, M.D.

OHIO.

J. Q. A. Hudson, M.D.

C. H. Tidd, M.D.

VIRGINIA.

John H. Claiborne, M.D.
F. Horner, Jr., M.D.
R. J. Preston, M.D.
J. E. Lockridge, M.D.
J. S. Wharton, M.D.
Wm. O. Owen, M.D.
Sam'l P. Ripley, M.D.
Benj. Blackford, M.D.
E. A. Drewry, M.D.
Rob B. Tunstall, M.D.
W. F. Barr, M.D.
L. B. Anderson, M.D.
J. M. Lazzell, M.D.

MISSISSIPPI.

C. B. Gallaway, M.D.
Edward Lea, M.D.
S. V. D. Hill, M.D.
E. T. Henry, M.D.
T. P. Lockwood, M.D.
Robert Kells, M.D.

ARKANSAS.

A. D. Hodge, M.D.
J. K. Hodge, M.D.
A. W. Hobson, M.D.

NEW JERSEY.

J. B. MATTISON, M.D.

### CORRESPONDING EDITORS:

Ely Van DeWarker, M. D., New York.
C. C. F. Gay, M.D., New York, *Surgeon of Buffalo General Hospital.*
M. H. Henry, M.D., New York, *Surgeon to N. Y. Dispensary, Dep't of Venerial and Skin Diseases.*
Benjamin Howard, M.D., New York.
James Tyson, M.D., Philadelphia, Pa.
W. W. Keen, M.D. Philadelphia.
Robert Robson, M D., Indiana.
A. Patton, M.D., Indiana.
John H. Weir, M.D., Illinois.
Thomas J Gallaher, M. D, Pennsylvania.
S. C. Busey, M.D., Washington, D. C.
Wm. R. Whitehead, M.D., Colorado Territory.

☞ The RECORD will be mailed between the 20th and 25th of each month.

